# Applying Text Analytics for Studying Research Trends in Dependability

**DOI:** 10.3390/e22111303

**Published:** 2020-11-16

**Authors:** Miriam Louise Carnot, Jorge Bernardino, Nuno Laranjeiro, Hugo Gonçalo Oliveira

**Affiliations:** 1 Polytechnic of Coimbra, Coimbra Institute of Engineering (ISEC), 3030-199 Coimbra, Portugal; m.carnot98@web.de; 2 CISUC, Department of Informatics Engineering, University of Coimbra, 3004-531 Coimbra, Portugal

**Keywords:** topic modeling, dependability, LDA, text analytics

## Abstract

The dependability of systems and networks has been the target of research for many years now. In the 1970s, what is now known as the top conference on dependability—The IEEE/IFIP International Conference on Dependable Systems and Networks (DSN)—emerged gathering international researchers and sparking the interest of the scientific community. Although it started in niche systems, nowadays dependability is viewed as highly important in most computer systems. The goal of this work is to analyze the research published in the proceedings of well-established dependability conferences (i.e., DSN, International Symposium on Software Reliability Engineering (ISSRE), International Symposium on Reliable Distributed Systems (SRDS), European Dependable Computing Conference (EDCC), Latin-American Symposium on Dependable Computing (LADC), Pacific Rim International Symposium on Dependable Computing (PRDC)), while using Natural Language Processing (NLP) and namely the Latent Dirichlet Allocation (LDA) algorithm to identify active, collapsing, ephemeral, and new lines of research in the dependability field. Results show a strong emphasis on terms, like ‘security’, despite the general focus of the conferences in dependability and new trends that are related with ’machine learning’ and ‘blockchain’. We used the PRDC conference as a use case, which showed similarity with the overall set of conferences, although we also found specific terms, like ‘cyber-physical’, being popular at PRDC and not in the overall dataset.

## 1. Introduction

With the increasing digitalization of our daily lives, more and more data are being generated, having their origin in very heterogeneous sources. A huge part of it is text written in natural language (e.g., social media), which brings in several complex challenges regarding extraction and synthesis for analysis (e.g., slang, sarcasm, multiple languages) [1]. Such challenges also obviously apply to more complex texts, like the ones in research papers. As the number and heterogeneity of research papers increases worldwide, it becomes increasingly difficult to obtain a synthetic image of the topics being investigated. Dependability is an established, but also expanding and heterogeneous, field of research, covering a large and dynamic number of subfields and touching a huge variety of Information Technology dimensions [2]. In addition, contributions to dependability are brought in by different research groups and, in multiple forms, which tends to make any analysis of the specific targeted subjects a complex problem. Thus, it is difficult to obtain a wide perspective image of exactly which topics of research are new, active, collapsing, or have been ephemeral.

In this work, we contribute with an analysis of the state of the art by using Natural Language Processing techniques and Topic Modeling, in particular the Latent Dirichlet Allocation (LDA) algorithm, to collect and synthesize information regarding topics of interest in well-known Dependability conferences. The main goal is to gain insight regarding topics of interest and understand how the field has developed in terms of the subjects of study, including active and decaying areas of research. The final analysis should also reflect an image of what has been achieved in this field, since its inception. Note that we do not intend to present a new model, but aim at using existing techniques in order to analyze a previously unexplored area.

Previous work has already analyzed different research fields [3,4,5,6]. However, to the best of our knowledge, such an application has not yet been done in the field of Dependability. It is difficult to obtain a clear overview of what Dependability covers, because the field has changed a lot over the years and it includes an increasing number of topics. In engineering, dependability is a branch of systems engineering. It describes the ability of a system to perform required functions under given conditions and thus can defensibly be trusted [7]. Mainly, it encompasses four components: reliability, maintainability, availability, and safety. System users can reasonably trust a dependable system. We analyze papers published in six well-known dependability conferences, namely: The IEEE/IFIP International Conference on Dependable Systems and Networks (DSN), the International Symposium on Software Reliability Engineering (ISSRE), the International Symposium on Reliable Distributed Systems (SRDS), the European Dependable Computing Conference (EDCC), the Latin-American Symposium on Dependable Computing (LADC), and the Pacific Rim International Symposium on Dependable Computing (PRDC), which we have selected for a more detailed analysis. We chose PRDC as a special focus because it is the only conference besides DSN for which all editions have been published on IEEEXplore and are therefore available. DSN is the main conference in the field of Dependability and makes up a huge part of our data set, so we could not imagine any major differences to the general overall analysis. Furthermore, PRDC is an emerging conference and the number of papers published has remained relatively constant over the years. We decided to analyze the conferences available on IEEEXplore because IEEE publishes the proceedings of most prestigious conferences in the field of dependability and also due to uniform access to data. DSN, ISSRE, and SRDS are all ranked A according to the CORE ranking (http://portal.core.edu.au/conf-ranks/), PRDC is ranked B, and LADC is not ranked. The dataset includes 5004 papers that were published since 1988 and until 2019 (i.e., all of the papers that are available online in IEEE Xplore and may be parsed). We aim to answer the following questions for the whole set of conferences, and then particularly for PRDC:

(RQ1) which were the most important terms and topics discussed?
(RQ2) which terms and topics represent recent trends and are possibly important for the future?
(RQ3) how did specific terms of interest developed throughout the years?

The results show the presence of expectable terms and topics in the global set of conferences (e.g., ‘fault tolerance’, ‘fault injection’), although they also highlight the decreasing usage of certain terms, like ‘software reliability’. We also observed a strong presence of security-related terms, like ‘vulnerability’, despite the general focus of the conferences being dependability. PRDC shows clear similarities with the global set of conferences, although we also found a stronger presence of specific terms, like ‘cyber-physical’. We also observed a recent trend on terms related with artificial intelligence (e.g., ‘machine learning’ ‘anomaly detection’) and on blockchain systems.

In short, the main contributions of this work are the following:

- the application of NLP techniques on a large dataset that includes titles, keywords and abstracts of research papers from the dependability field;
- the identification of the overall most frequent trends in the analyzed conferences and in PRDC separately;
- the identification of recent trends in the analyzed conferences and in PRDC separately; and,
- an analysis of the development of research trends over the entire time period and its interpretation.

The remainder of this paper is structured, as follows. In Section 2, we first provide some background knowledge, useful for understanding our methodology. In Section 3, we discuss similar work related to our topic before we present our approach in Section 4. In the following section, we present our results and the main findings. Finally, Section 6 discusses the conclusions and future work.

## 2. Background

This section should help the reader to understand basic NLP techniques that are used in our work in order to better comprehend the following sections. Among the techniques that are important for the understanding are topic modeling, especially the Latent Dirichlet Allocation Algorithm and text preprocessing. Topic modeling is a text mining technique that uses both unsupervised and supervised statistical machine learning methods for uncovering latent themes in large collections of documents, by grouping the words into word clusters, referred to as topics [8]. Text classification, on the other hand, is supervised, the classes must be defined in advance. In topic modeling, the possible topics are not known before. Topic Modeling recognizes which topics can be found in a document, whereas Text Classification only classifies the text in one class or another. In our case, we opted for Topic Modeling, because we did not know all of the trends that the analyzed papers might contain. Arguably, the most popular topic model is the Latent Dirichlet Allocation (LDA) [9], which may be used for finding new content, reducing the dimension for representing unstructured text or classifying large amounts of text. In LDA, each document is considered to be a mixture of latent topics. Each word in the document is assigned to a topic. Every topic is considered a mixture of words. These topics, whose number is determined at the beginning, explain the common occurrence of words in documents. In newspaper articles, for example, the words “euro, bank, economy” or “politics, elections, parliament” often appear together. These sets of words then each have a high probability in a topic. Words can also have a high probability in several topics. As explained by Blei et al. in [9], for each document *d* in a corpus *D*, LDA assumes the following generative steps: (1) choose length of document *N*; (2) choose topic proportion θ; (3) for each of the *N* words: (a) choose a topic *z*, (b) choose a word *w* from *p*, a multinomial probability conditioned on the topic *z*.

**Figure 1:**
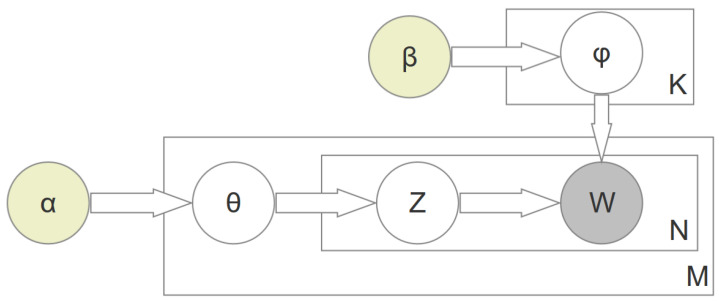
Latent Dirichlet Allocation (LDA) plate notation adapted from Blei et al. [9].

Figure 1 depicts the plate notation of the LDA algorithm. A plate notation is used in order to graphically depict variables that are repeating. The input parameters are α and β that represent the per-document topic distribution and the per-topic word distribution, respectively. The output are the words in *W*. The boxes are plates that represent repetitions. According to the parameter β, the word distribution ϕ for each topic in *K* is being determined. Parameter α influences the topic distribution θ for each document in *M*. The documents that are to be analyzed are depicted by the outer plate *M*, the word positions in a particular document are represented by the inner plate *N*. Each of these positions is associated with a selection of topics and terms. Based on θ, the topic assignment *Z* is being calculated for each specific term in *W*. The only observable variables are those words in *W*, which is why *W* is shown in grey. LDA only gives as output the terms that describe the different topics. All other variables are latent, which means that they are present, but not directly observed.

To maximize the benefits of text analytics, including LDA, text preprocessing is common. Sarkar [10] points out the importance of this step, which should include cleaning the whole dataset towards results that are easier to analyze (e.g., without meaningless words, spelling mistakes, etc.). One of the initial steps is tokenization, which splits text into tokens, its smaller meaningful units, often words. Yet, in order to better understand the meaning of a document, it might be useful to go beyond single tokens and analyze n-grams, which are sequences of *n* consecutive tokens. While unigrams are single tokens (n=1), *n* can be increased for longer sequences, such as bigrams (n=2), e.g., ‘machine learning’, or trigrams (n=3), e.g., ‘natural language processing’.

It is common to remove stop words, i.e., words that do not contribute enough to the meaning of a document. These are often functional words, like pronouns, articles, or prepositions, e.g., ‘you’, ‘the’, ‘at’. Preprocessing may further include lemmatization, which groups different forms of the same word in their lemma form, which carries the word meaning. Common rules include changing plurals to singulars (e.g., both ‘computer’ and ‘computers’ become ‘computer’) and using the infinite form of verbs (e.g., ‘write’, ‘written’ and ’wrote’ become ’write’). Stemming is an alternative to lemmatization, which represents words by their stem. Nevertheless, the stem is not always a valid word and it may not be possible to group irregular words (e.g., ‘wrote’ may become ‘wrot’ but ‘written’ ‘writ’). In some cases, additional steps may be taken, e.g., for expanding contractions, correcting spelling mistakes, or eliminating repeated characters.

## 3. Related Work

We have identified four main groups of research that are related to ours, namely works that: (i) use LDA to analyze scientific papers and other documents over the years; (ii) use Topic Modeling to filter out latest news and articles in social networks; (iii) describe the importance and use of text analysis in general and Topic Modeling in particular; and, (iv) use topics and paper citations to overview a research area.

### 3.1. Lda to Analyze Scientific Papers and Other Documents over the Years

Hall et al. [11] analyzed historical trends and developments over time in the field of Computational Linguistics while using LDA. They focus on three well-known conferences in the 1978–2006 period and, among other trends, discovered a general increase in application research and a decrease in semantics research. They also note that, over time, research trends became more similar in the three conferences, whereas the general diversity in research trends is expanded. Their work relied exclusively on unigrams, which usually have limited informative value. This is why we included bigrams in our analysis, and focused mainly on them.

To discover trends in Artificial Intelligence (AI), Saari [4] analyzed NIPS conference papers from 1987 to 2018. The LDA topic model was compared to a model that was based on semantic word vectors (sHDP), and outperformed it in both quality and coherence. As in our work, Saari used the Gensim library in Python for preprocessing. Finally, three different epochs in AI research were found (neuroscience era, algorithmic era, and deep learning era) and a tendency from neural information processing towards deep learning. In contrast to our research, Saari used full publications, not only titles and abstracts.

In order to predict upcoming research trends, Francopoulo et al. [3] tested various algorithms on a large dataset of papers that were published in the main scientific events on Natural Language Processing. Briefly, they pre-processed the textual data and learned a time-series from past events to compare the accuracy of different prediction algorithms. Authors discuss that the gap between prediction and observation grows larger the further the prediction lies in the future, e.g., a prediction for the coming year might be very accurate, whereas a prediction for the year 2030 will most likely be incorrect. They also admit that applying this method to another domain would require the involvement of a specialist, e.g., for collecting stop words and identifying synonyms.

Moro et al. [5] examined 219 journal articles in the field of banking and business intelligence while using text mining techniques, including LDA. Their aim was similar to ours: identifying research directions in the field of interest. Yet, in contrast to our work, they are only interested in recent topics, not in their evolution over time. They also showed in which topics future research is needed in the field.

Griffiths et al. [6] introduce an algorithm to draw conclusions from the LDA model results, while providing quantitative measurements that allow for the subject matter of documents to be identified, changes in topics to be tracked over time, and similarity between works of different fields. The algorithm was applied first to a small dataset and then to abstracts from the Proceedings of the National Academy of Sciences (PNAS) conference between 1991 and 2001.

Saari [4] and Griffiths et al. [6] both compare a single conference, whereas we compare six and, thus, have a larger dataset, which should make the study more meaningful.

Wang et al. [12] developed a new model that considers both the co-occurrence of the words and temporal information in order to produce more distinct topics than regular LDA. Normally, time must be sliced (e.g., in years, like we did), but, in their argumentation, it is not reasonable, because some events took place for a longer time and others for a shorter. When tested in real-world datasets comprising periods with significantly different durations (nine months, 17 years, 21 decades), the model showed to be good at localizing topics and trends in time. Because our goal is to give a general overview of the trending research topics in the dependability field over time, the division into years seemed to be adequate for us.

### 3.2. Filtering Latest News in Social Networks

The most common use of Topic Modeling was probably to show the latest trends in social networks. In this context, Lau et al. [13] introduced a novel topic model in order to analyze emerging on-line trends on the Twitter social network. The evolution of topics is calculated on every new update to always obtain the most recent events discussed. The developed model adds previously unseen words to the vocabulary and removes words that fall under the threshold not to grow in size over time. They tested the model on synthetic datasets with a single new event, then several emerging events, and finally live tweets in London and New York. In this paper, we only want to analyze the change of topics in the past on a predefined dataset, so the regular LDA model is appropriate.

Abd-Alrazaq et al. [14] used the LDA algorithm in order to identify the main topics of the Twitter posts related to the COVID-19 pandemic. Like us, they opted for Lemmatization instead of Stemming and computed bigrams and adding them to the text. Besides the text, they also made use of the metadata of the tweets, e.g., number of likes and retweets, number of followers, to figure out the popularity and interaction of each topic. Abd-Alrazaq et al. found that the highest mean of likes was for tweets about economic loss (13.413), while the lowest was connected to travel band and warnings (3.94). They conclude that the public health system must be more present and active in social media to reduce the spread of inauthentic information.

Hong et al. [15] studied the use of Topic Modeling in the social network Twitter. They present different schemes for training topic models and then compare those regarding quality and effectiveness. They also tested the schemes and found that the results were better by training the model on aggregated data. They show that the quality of the results of a trained topic model is strongly connected to the length of the documents.

### 3.3. Describing Text Analysis and Topic Modeling

On the utility of Topic Modeling, Jelodar et al. [8] survey past work, show in which fields it can be applied (e.g., Politics, Medicine, Geography, and Software Engineering) and where there are gaps in the research done so far. They also describe very shortly how to use different extensions of LDA and what common tools and datasets are available.

Song et al. [16] offer several topic and keyword re-ranking methods that were tested in different experiments using two datasets. The order of words and topics in the LDA is difficult to understand for users, as some words might appear in several topics and topics are randomly ordered. Accordingly, for the LDA results to be clearer, the algorithms re-weight the native weight of each term while using scores and re-rank the topics based on different criteria.

Liu et al. [17] give an overview about Topic Modeling specifically in the field of Bioinformatics and where it is being applied. They found that Topic Modeling proves very useful for interpreting biological data. Though, it has not been applied widely using biological data and needs further research.

Alghamdi et al. [18] discuss not only different methods of Topic Modeling (LSA, PLSA, LDA, and CTM), but also different Models in Topic Modeling such as topic over time (TOT), dynamic topic models (DTM), and many more. For each of the Methods and Models they presented their characteristics and limitations, and also the differences between them. This survey can be very useful for deciding which method and model to choose for a specific use case.

### 3.4. Use of Topics and Citations to Overview a Research Area

Finally, there also exists works that try to link collected knowledge and capture the essence of a domain. For instance, Dietz et al. [19] aim to facilitate a quick overview of an unknown research field by measuring the influence of citations in a given work. They perform experiments using six different approaches, some of them based on LDA, and show that differences among them are only significant for a high number of topics (30 or 50). For the experiments, they use title, abstract, and citation information from a set of research papers. Finally, domain experts evaluated the accuracy of the generated topics. In contrast to our work, more cited papers have a greater influence on the outcome of the LDA model. Our work pursues a slightly different goal, which is to analyze trends where all of the papers should weight the same. Citations are always made after the paper was published, sometimes several years, but we want to find all the topics up to today.

Sriurai et al. [20] propose an LDA-based topic-model that helps in finding related articles in Wikipedia Selection for Schools. Every article can be regarded as a probability distribution over the generated topic set. Therefore, two similar topic distributions show a conceptual relation between those articles. They run LDA on the titles and anchor-texts of the articles, and compute article similarity with the dot product between their topic probability vectors. However, the topic similarity must always be considered subjectively. In our work, we focus less on the connection between single documents and more on the interaction of the collection as a whole.

Jo et al. [21] detect topics that are based on the assumption that there is an interaction between the distribution of a topic-specifying term and the link distribution in the citation network. They introduce a topic score measure that measures the probability of graph connectivity for each term, i.e., two documents are more connected if they both contain a topic-relevant term. They tested the algorithm on two online collections of research papers. The more relevant high scoring topics are less closely linked than the smaller but more specific topics. Similar to our work, they consider bigrams and examine both titles and abstracts.

Mann et al. [22] rely on topic models for providing users of online libraries with an overview of an unknown subject field, in order to draw their attention to important documents, and to reveal connections between fields and sub-fields. They ran a phrase-based topic model on a collection of Computer Science publications from the Rexa digital library (http://rexa.blogspot.com). They pointed out that only the citation count of a paper does not expose the significance of this paper in the field. Just like us, they run the model for both unigrams and bigrams. This paper supports our work in the approach of not considering the number of citations, as it is more related to popularity than to the significance of topics.

In this paper, we designed a study for discovering the changes in topics over time in different dependability research conferences. Text analytics techniques have not previously been applied to this domain, to the best of our knowledge.

## 4. Methodology

In this section, we present our approach to analyze topic trends in well-established dependability conferences. In practice, we are interested in understanding which dependability topics are new or active, decaying, or have been most prominent. Our analysis goes through the following steps:

(1) data acquisition and preparation;
(2) exploratory analysis of frequent author keywords for each year;
(3) topic Modelling with LDA; and,
(4) analysis of frequent terms in LDA topics for each year.

We began with the acquisition and preparation of the data, where we aimed at collecting information regarding the following well-known dependability conferences:

- International Conference on Dependable Systems and Networks (DSN formerly FCTS);
- European Dependable Computing Conference (EDCC);
- International Symposium on Software Reliability Engineering (ISSRE);
- International Symposium on Reliable Distributed Systems (SRDS formerly RELDIS and RELDI);
- Latin-American Symposium on Dependable Computing (LADC); and,
- Pacific Rim International Symposium on Dependable Computing (PRDC).

The IEEE Xplore website [23] was our data source. Because it gathers the proceedings of the majority of the editions of the above mentioned conferences, we scraped it to extract metadata. Table 1 details our dataset, including the number of papers published per year and conference.

The dataset holds metadata regarding a total of 5004 papers, published between the year 1988, the first edition of DSN (at the time, known as FCTS) and 2019. Most of the conferences were established later than 1988 and some did not have an edition every single year. Additionally, fourteen editions of these conferences were not included because their proceedings were published by a different publisher and were, thus, not available from IEEE Explore, namely: four editions of EDCC (1994, 1999, 2002, 2005), three editions of LADC (2003, 2005, 2007), ISSRE 1990, and the six editions of SRDS before 1988.

For each paper identified, we used JSOUP (https://jsoup.org/), a web scraper tool, for extracting the Digital Object Identified (DOI), title, abstract, and also “keywords”, which IEEE Xplore separates in the following four types: author keywords (i.e., assigned by the authors); IEEE keywords that come from the IEEE taxonomy (https://www.ieee.org/content/dam/ieee-org/ieee/web/org/pubs/taxonomy_v101.pdf), which includes different scientific fields with a maximum of three sublevels; INSPEC controlled indexing keywords (i.e., listed in the Inspec Thesaurus) (http://images.webofknowledge.com/WOKRS59B4/help/INSPEC/hs_controlled_index.html); and, INSPEC non-controlled indexing keywords (i.e., assigned by INSPEC indexers, but in free-language words and phrases). It is important to mention that we eliminated extraneous documents that were found in the proceedings from the acquisition, namely those whose title contained the following words: committee, organizers, organization, abstracts, forum, subcommittee, message, workshop, keynotes, publisher, sponsors, acknowledgments, and symposium. This elimination was necessary in order to avoid documents in the dataset that are not actually research papers.

Before running LDA, we performed an exploratory analysis of common author keywords in the dataset. Author keywords are easy to acquire and very meaningful, because they were set by the authors themselves, even though they are, unfortunately, only available for papers since 2004. We decided not to put a special focus on the INSPEC and the IEEE keywords as they proved less meaningful than the author keywords (e.g., ‘proposals’, ‘hardware’, ‘frequency’), thus their analysis would lead to less particular insights. We mapped each different keyword present in the dataset to the respective total number of occurrences in the whole dataset. We also retrieved the count per year and per conference, for further analysis.

It is worthwhile mentioning that we also tried to identify meaningful terms by examining the most common terms (i.e., unigrams and bigrams) that were retrieved from the titles and the abstracts, but the results were not very helpful. For example, the most frequent unigrams in titles and abstracts were: ‘software’, ‘system’, ‘fault’, ‘data’, ‘network’, ‘model’ and ‘reliability’. The most frequent bigrams found were, for example, ‘large scale’, ‘real time’, ‘computer science’, or ‘case study’, which we found to be very generic, making it difficult to obtain helpful insights.

In order to identify the research topics in the identified papers, we then run the LDA algorithm in the text of the abstracts and titles (as a whole) per year. As we mention in the Background section, LDA is the most popular algorithm for topic modeling. We were particularly interested in understanding the topics of relevance per year (and across all conferences). LDA is based on a repeated random selection of text segments, whereby the statistical accumulation of word groups is recorded within these segments. Thus, the algorithm calculates the topics of the text collection, words that belong to each topic, and their salience. LDA offers better quality and coherence than other available topic modeling algorithms, as shown by Saari [4].

Yet, before actually running LDA, we pre-processed the input data, which is especially important when running this algorithm [10]. For this, we used the Natural Language Toolkit (NLTK) [24] and applied the preprocessing techniques (see Background section) that we deemed to be appropriate for our case, also considering related work [10]. First, we tokenized all the texts and converted the tokens to lowercase. We removed numbers but not words containing numbers to not exclude Numeronyms (e.g., ‘industry4.0’, ‘3G’, ‘AIS256’), as they may carry relevant information. We also removed all words that are one single character long, as they are not useful. We then lemmatized all the words. After this, we used the gensim [25] Python library in order to compute bigrams for each paper (i.e., for each abstract and title), which becomes associated with both its respective single-word terms and combined tokens (e.g., if the text contains ‘[…] software reliability […]’ the output will contain the terms ‘software’, ‘reliability’ and ‘software_reliability’). Bigrams tend to be more specific and, in that sense, they are more informative (e.g., ‘software reliability’ is more informative than ‘sofware’). Additionally, some common unigrams are very hard to interpret when not associated with a second word (e.g., ‘base’, ‘core’, ‘case’, ‘solution’). We only considered bigrams that appear at least five times, as we observed that a lower number tends to produce meaningless combinations of terms (e.g., ‘sense_to’, ‘look_reveals’).

An important step after adding bigrams is removing stop words. Besides common English stop words, available in NLTK, we added a number of selected stop words that we found to be meaningless in our context, as they are not specific to the dependability domain (e.g., ‘year’, ‘conference’, ‘author, ‘approach’). Some other techniques were not necessary in our case, for instance, expanding contractions, correcting spelling mistakes, or eliminating repeated characters, because colloquial language and spelling mistakes are rather uncommon in research papers. In order to normalize words, we opted for lemmatization instead of stemming, because the results of the latter are harder to interpret, especially with bigrams.

Regarding the configuration of the LDA model, we performed an exploratory analysis of several different parameters, before we committed ourselves to the following. In LDA, the corpus is always represented as a bag-of-words of the documents, which means that the order of the words does not matter. Even though we experimented with a smaller and larger number of topics, we decided to generate 20 topics, both because different numbers seemed to produce less informative topics and because 20 is a commonly used number for this purpose [8]. There are no precise instructions on how to set the value K optimally for the topics to be generated, since LDA is an unsupervised method. Various researchers have developed approaches for determining K in the best possible way [6,26]. However, other research recommends not to rely on these methods, but to check the quality of the topics with different numbers [27].

We opted to represent each topic with 20 words, because this allows for a more detailed characterization of the topic. We empirically noted that a low number of words tends to make topic labeling harder, while a high number tends to add irrelevant terms. We set the number of passes to ten, which means that the model is trained ten times on the entire corpus. This number needs to be high enough for all of the documents to converge enough, so that there are not too many diverse topics (in the worst case one topic for each document). On the other hand, if the number of passes is too high, we would get twenty very similar topics because they would become increasingly convergent. As output, we obtain a list of topics sorted in descending order from the most frequent topics to the less assigned topics.

After this, we wanted to analyze the terms in the LDA-generated topics. In total, we considered a timespan of 32 years, which, if we multiply by 20 topics, equals 640 topics. As each topic consists of 20 terms (i.e., 20 words), we end up with 12,800 words, including possible duplications. Note that, due to the nature of the LDA algorithm, which aims at uncovering the most meaningful terms for the analyzed documents, we were expecting to obtain a very different set of terms than the author keywords, which makes them interesting to analyze. We then selected the most frequent unigrams, namely those with at least ten occurrences, when considering all of the terms from all generated topics (i.e., all the years).We also selected the most frequent bigrams (at least two occurrences), but we did this separately, as we observed that they would rarely appear in the list of all the most frequent terms. Nevertheless, we still had to eliminate some less informative terms manually, e.g., ‘number_of’, ‘paper_we’, ‘such_a’, ‘show_that’.

Finally, we relied on the outcome of this process for analysing how the popularity of the key terms that were generated by LDA changed over time. In particular, we were interested in analyzing the frequency of LDA terms (also author keywords) over the whole timespan considered (i.e., the 32 years), but also focusing on the last three years, with the goal of identifying recent trends.

## 5. Results

In this section, we present the results of the analysis performed according to the methodology and considering two main views: (a) global view of the whole dataset; and, (b) particular view of the PRDC conference. The analysis goes through the following parts:

1. keyword analysis;
2. identification of the top ten LDA topics; and,
3. analysis of handpicked LDA terms across time.

### 5.1. All Conferences Analysis

In this section, we analyzed the papers that were gathered from all six conferences. The analyzed time frame is from 1988 to 2019, i.e., 32 years. Some conferences published more papers than others, e.g., DSN or ISSRE. This of course also means that these conferences have a stronger influence on the analysis. This chapter’s goal is to give a general overview of the dependability field regardless of the conferences.

We start with the identification of common keywords, when considering all conferences. Table 2 shows the top ten most frequent terms, used as author keywords in all conferences and the top fifteen most frequent terms in the last three years of the remaining words.

As we can see in Table 2, regarding the whole analysis period and, despite the focus of the conferences being dependability, there is a large number of papers using ‘security’ as a top keyword. The results also show the presence of expectable keywords, like ‘fault injection’, ‘fault tolerance’, or ‘distributed systems’. It is worthwile noting the presence of ‘cloud computing’ (as a domain of application) and also ‘machine learning’, which is likely related with the known recent trend in this area [28]. If we only consider the last three years, the results mostly confirm the common sense that suggests there is additional focus in topics, like blockchain systems, Internet of Things, and privacy.

In Table 3, we present the top ten topics identified by LDA out of twenty produced by the LDA algorithm for all papers present in our dataset. The topics have been ordered by LDA, according to the number of papers they describe and we also show the specific terms composing each topic (limited to the top 10 terms, for space reasons). LDA orders these terms by their salience, which indicates the importance of a certain term to the topic. It is difficult to describe each topic in a title as the topic looses meaning by reducing it to one single term. When we tried to describe the topics, there was repetitions, so that we decided to leave them without titles.

Based on the LDA topics, we separately identified the most popular unigrams and bigrams, i.e., those appearing more frequently in the whole set of generated topics. Table 4 presents the results of this identification. Even though the frequencies for this table were counted on the twenty topics for each single year. We can see a clear connection between this table and the topics that were generated for the papers for all years shown in Table 3. For example, the term ’performance’ is the most frequent term for the whole period when considering the topics for each year and it is also the most frequent in our presented LDA topics for all years.

We found the unigram list to be quite generic (e.g., ‘performance’ may have different meanings, depending on the context) and, as such, we found the bigram information much more informative. In this latter case, we find the top three terms clearly exposing the nature of the conferences (i.e., ‘fault tolerance’, ‘software reliability’, ‘fault injection’), with the remaining showing areas of application (e.g., ‘distributed system’, ‘operating system’) and terms that are likely to be related with the evaluation of proposals (e.g., ‘test case’, ‘case study’, ‘large scale’). We also note the presence of terms, like ‘soft error’ and ‘safety critical’, which further characterize the work being published.

The analysis of the last three years unigrams again yields little useful information, although we may highlight ‘cloud’, ‘bug’, and ‘safety’. However, if we analyze the last three years bigrams, then it is worthwhile mentioning the obvious presence of ‘machine learning’, ‘smart contract’ (i.e., blockchain code), and also ‘anomaly detection’ (which is very much related with machine learning). The remaining terms are quite diverse, but we highlight ‘static analysis’, which would not be obvious presences in this top recent bigrams.

There are several terms that have a simultaneous presence in the LDA topics and keywords, which emphasize the presence of certain subjects of interest. When considering the whole period, this is the case of ‘security’, ‘fault tolerance’, ‘fault injection’, and ‘distributed systems’. If we look at the last three years, we also find ‘safety’ and ‘static analysis’ being used in keywords and appearing in popular LDA terms. Then, we find a few more terms that do not exactly map (as words), but are semantically related. This is the case of ‘cloud computing’ and ‘cloud’, and also ‘smart contract’ and ‘blockchain’. It is also worthwhile mentioning that we found three terms that have presence as keywords in the overall period, but are found to be popular only in the LDA terms for the last three years. This is the case of ‘cloud computing’, ‘anomaly detection’, ‘machine learning’, with LDA marked as being particularly interesting for the recent years.

**Figure 2:**
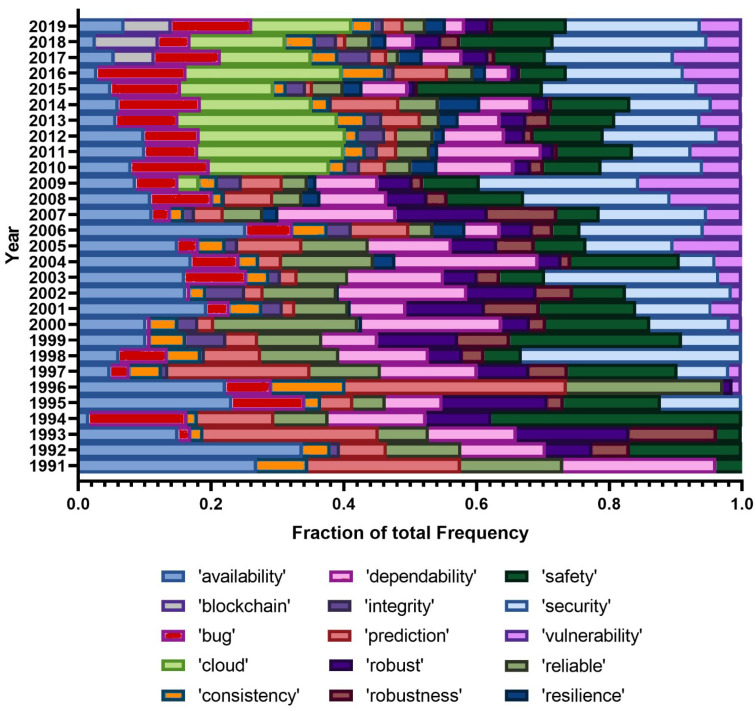
Selected unigrams from the LDA results, for all conferences.

**Figure 3:**
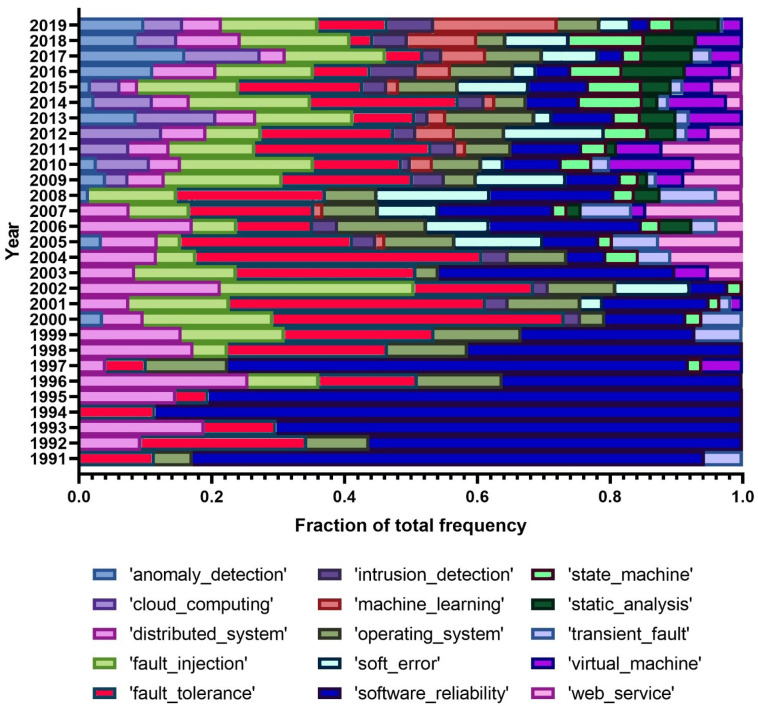
Selected bigrams from the LDA results, for all conferences.

After analysing popular terms, we handpicked a few terms of interest and analyzed their popularity across the whole period, i.e., from the beginning of the conferences. Figure 2 and Figure 3 show the selected unigrams and bigrams and their relative popularity across time. We excluded the years 1988–1990 as our handpicked terms only rarely or not at all appeared in those years, due to a very small number of papers in the first years analyzed.

Figure 2 very clearly shows that the use of the term ‘dependability’ is losing expression and this is very likely due to the fact that, nowadays, although the focus is obviously still dependability, the aspects being researched tend to be finer and much more specific, whereas in the early years of the conferences dependability in itself, as a whole, was a sufficiently novel and detailed term to be used in abstracts. There is a noticeable presence of ‘bug’ until recently, with the same happening with ‘safety’ and ‘security’. The term ‘vulnerability’ accompanies ‘security’, although with a lower relative frequency. More recently, we find ‘resilience’ (with a relatively low frequency) and ‘cloud’, associated with the advent of cloud computing. Additionally clear is the recent presence of ‘blockchain’, which is in line with its known recent popularity.

As Figure 3 shows, we find ‘fault injection’ as currently the most popular term and we see that, despite some variation, its popularity followed a global positive trend. It is interesting to note the strong decrease of the use of ‘software reliability’ as terms of relevance in the abstract. Similarly, there is a trend with the term ‘web services’ initiating in 2003 but closing in 2016. At the same time, we see ‘operating system’ with a fairly equal presence throughout the years, with the same happening with ‘soft error’ although it has been used only from the beginning of the 2000’s. ‘Distributed system’ and ‘fault tolerance’ tend to be used and are popular, although their relative frequency has decreased. Recent and rising terms are ‘machine learning’ and ‘anomaly detection’, which clearly show the interest of the community in artificial intelligence topics and the confluence of dependability with these fields.

### 5.2. PRDC Conference Analysis

We now take a closer look at the IEEE Pacific Rim International Symposium on Dependable Computing (PRDC) and begin with the identification of common keywords for PRDC, which we summarize in Table 5.

Table 6 presents the top ten topics identified by LDA for PRDC, and their first ten associated terms. We can see that the topic around security-related terms appears in both the topics of all the conferences and only for PRDC in one of the first places. Topic number 7 in PRDC could be related to networks, also topic number 6 in the overall analysis probably describes papers that are connected to networks. The term ‘dependability’ appears as first term in the first topic for PRDC, which clearly shows its importance to the conference, whereas we can find the same term in only topic number 10 for all of the papers in the dataset.

As previously noted, we calculated the most popular unigrams and bigrams present in the LDA topics, which are shown in Table 7.

Once more, the unigrams revealed again to be little informative, with the exception of a few terms, like ‘security’ or ‘safety’. Still, it is interesting to notice network-related terms at the top two positions. In the bigrams, we find that half of the top ten terms match those that we had previously observed for the whole dataset, namely ‘fault tolerance’, ‘fault injection’, ‘software reliability’, ‘soft error’, and ‘safety critical’. We also find ‘fault detection’ and ‘error detection’ to be strongly associated with PRDC, and also ‘web service’, which is likely the consequence of the time period and number of editions of PRDC being smaller.

When considering the last three years, we may emphasize ‘attack’, ‘cloud’, ‘iot’, ‘blockchain’, or ‘privacy’, which are the most descriptive unigrams in the list. The bigrams show the interesting case of ‘cyber physical’ being the top term (not found in the top terms for the whole dataset). We then find the expected case of ‘machine learning’, and then ‘anomaly detection’ and also cloud related terms like ‘cloud computing’. The frequency of the top six terms is much larger than the remaining six, which, in this sense, should be considered to be less informative.

**Figure 4:**
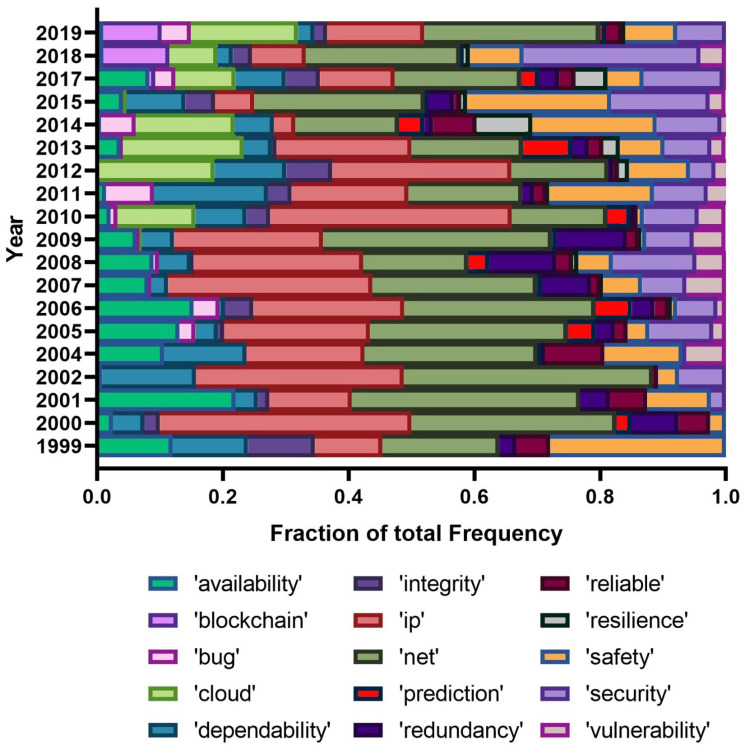
Selected unigrams from the LDA results, for the PRDC conference.

**Figure 5:**
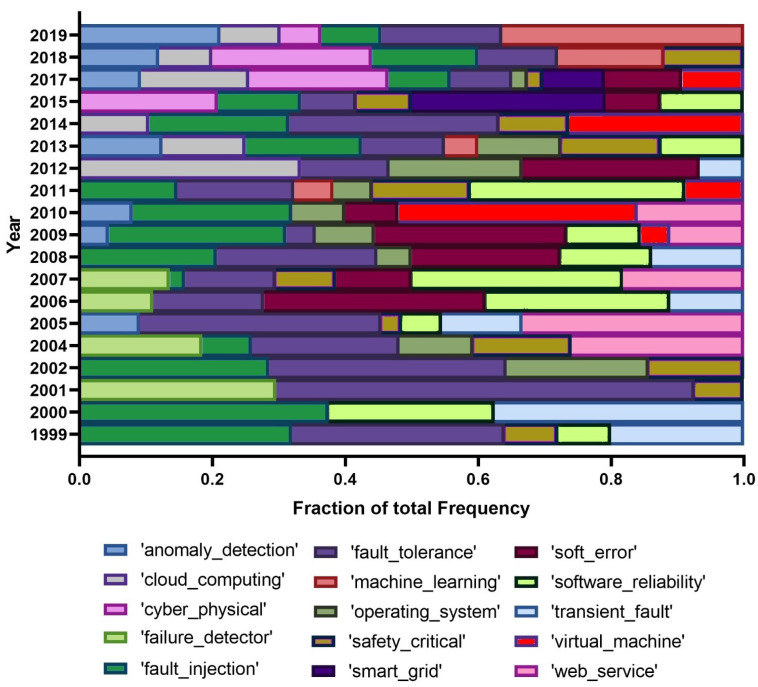
Selected bigrams from the LDA results, for the PRDC conference.

Finally, we handpicked a few terms of interest from PRDC and analyzed their popularity for the time period of this conference. Figure 4 and Figure 5 hold a visual representation of the relative prevalence of these terms over time.

Regarding Figure 4, there are a few cases of interest, namely the relatively strong presence of ‘ip’ and also ‘net’ throughout the time. The topics ‘safety’ and ‘security’ have gained popularity over time. ‘Dependability’ used to be a frequent term in the early years of the conference, but it is now much less important. The presence of the term ‘vulnerability’ is also noticeable, although its popularity globally dropped over time. The term ‘bug’ closely follows the pattern of ‘vulnerability’. The presence of resilience is scarce throughout and, among the selected terms, ‘cloud’ has seen a boost since 2009 and ‘blockchain’ also, but since the last three years. Similar to the whole dataset, ‘availability’ lost interest over the years. Figure 5 holds the bigrams’ popularity representation. We must emphasize the general presence of ‘fault tolerance’ and ‘fault injection’ as frequent terms. Additionally, we noticed the case of ephemeral terms, namely, ‘smart grid’, ‘web service’, or ‘failure detector’. ‘Machine learning’ and ‘anomaly detection’ show positive trends, in line with what was observed for the whole dataset. Finally, we found ‘operating system’ to not have a presence as strong as was obtained in the whole dataset.

## 6. Conclusions and Future Work

In this work, we analyzed 5005 research papers from six different well-established dependability conferences while using the Latent Dirichlet Allocation (LDA) algorithm. It was possible for us to identify the most important topics of the analyzed conferences and to interpret some of them. Over the years, the Dependability Conferences have adapted to the prevailing IT trends. The number of papers published has increased with time. The most popular topics in the analyzed conferences were Fault Tolerance, Software Reliability, Fault Injection, but also Security. Contemporary trends include machine learning, anomaly detection, Internet of Things, blockchain systems, and especially clouds. We took a closer look at the PRDC conference, which showed a few resemblances with respect to the whole dataset (e.g., ‘safety’ and ‘security’ are generally present, ‘machine learning’ is a recent trend), but also a few clear differences (e.g., ‘operating system’ is a less frequent term, ‘cyber physical’ is associated with PRDC as the top term, but it is not on the top ten for the whole dataset). We could observe that over time the classical topics merge with current topics, such as artificial intelligence, and that a greater diversity of topics has developed over the years. The conferences are becoming more diverse and also deal with topics that would not necessarily be classified as belonging to the Dependability field in the classical sense. The current trends in the field of Dependability largely coincide with the prevailing trends in computer science in general.

However, there are a few threats to the validity of this work. A first clear point is the lack of editions of the conferences by publishers other than IEEE (i.e., 14 editions missing in total). Even so, the dataset includes the large majority of the works. A related aspect is with the number of conferences, which could be extended, although we believe these are considered the most strongly connected with dependability. We must mention that several operations had to be done manually, such as selecting stop words and terms for elimination. The LDA algorithm chooses the most meaningful terms of a certain input, but these may not always be the terms that are the most meaningful for the domain. Without this manual elimination the analysis would loose a lot of its meaning, but at the same time it could add bias to the results. This manual elimination was checked by a second researcher, in order to assure no particularly important information would be lost as a direct consequence of this step.

We analyzed selected terms, which may add bias to the discussion. Despite this, the selection was made with agreement by two researchers, as a way of reducing any individual bias. Finally, we have chosen to only analyze the author keywords, whereas the other types of keywords could also provide relevant information about the development of research trends. Again, this decision was taken after analyzing the different types of keywords, and the decision was taken over the set we found to be more descriptive.

Our work helps to provide a clearer view of the research being published in well-known dependability conferences. It may help researchers in selecting research topics of interest and clearing decaying topics.

As future work, we intend to predict future research trends by training different models with the data. We also intend to explore the meaning of the terms by using deep semantic representations, which allow for bringing further information and improving understanding regarding research trends.

## Figures and Tables

**Table 1:** Number of documents in the dataset, per target conference, and year.

	DSN	EDCC	ISSRE	LADC	PRDC	SRDS
1988	50					15
1989	61					12
1990	51					11
1991	53		22			14
1992	52		28			20
1993	65		29			8
1994	40		33			14
1995	50		38			17
1996	38		34			17
1997	38		41			19
1998	44		42			63
1999	38		27		26	47
2000	59		26		19	19
2001	47		32		46	28
2002	85		27		35	44
2003	73		36			32
2004	85		39		42	31
2005	81		32		54	20
2006	57	19	43		56	38
2007	82		26		57	27
2008	58	19	56		49	28
2009	66		21	21	54	30
2010	66	24	42		42	46
2011	51		27	19	46	30
2012	51	24	39		16	71
2013	68		45	15	48	21
2014	82	29	31		31	45
2015	50	23	48		39	33
2016	58	27	46	26		39
2017	55	27	35		49	32
2018	62	29	23	19	43	32
2019	54	31	41	25	46	47
Total:	1870	252	1009	125	798	950

**Table 2:** Most frequent author keywords in all years and in the last three years and their frequency.

All Years	Freq	Last 3 Years	Freq
‘security’	125	‘security’	49
‘reliability’	116	‘fault tolerance’	32
‘fault tolerance’	116	‘blockchain’	26
‘fault injection’	79	‘machine learning’	24
‘dependability’	77	‘internet of things’/‘iot’	22
‘distributed systems’	48	‘reliability’	20
‘cloud computing’	45	‘anomaly detection’	20
‘anomaly detection’	39	‘fault injection’	18
‘software reliability’	36	‘privacy’	17
‘machine learning’	36	‘dependability’	16
		‘cloud computing’	15
		‘distibuted systems’	13
		‘safety’	12
		‘consensus’	11
		‘android’	11

**Table 3:** Top ten LDA topics for all papers in the dataset.

Topic 1
server, web, cloud, client, architecture,
user, platform, environment, performance, request
**Topic 2**
vulnerability, security, robustness, hardware, study,
web, exception, user, static, runtime
**Topic 3**
case, study, coverage, test_case, quality,
empirical, requirement, source, case_study, input
**Topic 4**
hardware, processor, memory, bit, redundancy,
transient, overhead, architecture, performance, chip
**Topic 5**
safety, design, verification, specification, property,
framework, present, checking, safety_critical, support
**Topic 6**
performance, routing, log, peer, packet,
mobile, traffic, wireless, user, communication
**Topic 7**
replication, performance, storage, transaction, replica,
availability, consistency, update, file, multicast
**Topic 8**
algorithm, number, byzantine, faulty, optimal,
case, consensus, bound, processor, gt
**Topic 9**
software_reliability, distribution, metric, estimate, defect,
release, rate, number, product, prediction
**Topic 10**
availability, dependability, markov, repair, stochastic,
evaluation, net, aging, rejuvenation, performance

**Table 4:** Most frequent LDA terms for the whole period of analysis and last three years, for all conferences.

All Years	Freq	Last 3 Years	Freq
‘performance’	2752	‘attack’	564
‘log’	2438	‘security’	502
‘algorithm’	2316	‘performance’	454
‘design’	2052	‘source’	387
‘experiment’	1920	‘log’	364
‘attack’	1875	‘cloud’	348
‘security’	1873	‘safety’	262
‘source’	1860	‘user’	237
‘effect’	1733	‘device’	252
‘replica’	1706	‘evaluation’	2029
		‘bug’	222
		‘injection’	214
		‘framework’	202
		‘smart’	200
		‘environment’	200
‘fault_tolerance’	539	‘fault_injection’	60
‘software_reliability’	458	‘machine_learning’	51
‘fault_injection’	360	‘anomaly_detection’	44
‘case_study’	281	‘case_study’	39
‘test_case’	278	‘large_scale’	38
‘large_scale’	246	‘test_case’	38
‘distributed_system’	239	‘safety_critical’	33
‘operating_system’	233	‘smart_contract’	30
‘soft_error’	169	‘cloud_computing’	29
‘safety_critical’	169	‘static_analysis’	29
		‘soft_error’	28
		‘distributed_system’	25
		‘operating_system’	25
		‘data_center’	23
		‘fault_tree’	23

**Table 5:** Most frequent author keywords in all years and in the last three years in the Pacific Rim International Symposium on Dependable Computing (PRDC) conference and their frequency.

All Years	Freq	Last 3 Years	Freq
‘fault tolerance’	45	‘fault tolerance’	10
‘reliability’	30	‘security’	8
‘dependability’	27	‘blockchain’	6
‘security’	22	‘internet of things’	6
‘fault injection’	16	‘machine learning’	5
‘availability’	11	‘reliability’	5
‘safety’	9	‘dependability’	4
‘software reliability’	8	‘cyber physical systems’	4
‘distributed systems’	8	‘fault injection’	3
‘consensus’	8	‘distributed systems’	3
		‘anomaly detection’	3
		‘distributed algorithms’	3
		‘software testing’	3
		‘mesh array’	3
		‘cloud computing’	2

**Table 6:** Top ten LDA topics for the PRDC papers.

Topic 1
dependability, communication, algorithm, resource, availability,
consensus, scheduling, replication, deadlock, performance
**Topic 2**
disk, parity, number, array, organization,
software_reliability, point, performance, behavior, rate
**Topic 3**
security, dependability, cyber, grid, performance,
iot, threshold, blockchain, attack, power
**Topic 4**
injection, fault_injection, attack, hardware, design,
algorithm, architecture, malware, flow, device
**Topic 5**
availability, memory, cloud, virtual, performance,
routing, bit, communication, sub, machine
**Topic 6**
circuit, controller, cloud, decision, hardware,
device, algorithm, storage, resource, cost
**Topic 7**
algorithm, delay, route, memory, routing,
dependable, research, chip, study, machine
**Topic 8**
algorithm, different, location, performance, study,
scan, multicast, pattern, wireless, mobility
**Topic 9**
array, processor, core, circuit, element,
overhead, hardware, spare, security, disk
**Topic 10**
study, algorithm, design, attack, overhead,
security, flow, hardware, scan, architecture

**Table 7:** Most frequent LDA terms for the whole period of analysis and last three years, for the PRDC conference.

All Years	Freq	Last 3 years	Freq
‘net’	881	‘net’	166’
‘ip’	724	‘security’	111
‘algorithm’	562	‘attack’	105
‘log’	421	‘ip’	84
‘performance’	420	‘cloud’	78
‘memory’	335	‘algorithm’	64
‘security’	331	‘iot’	62
‘design’	330	‘log’	60
‘safety’	295	‘safety’	51
‘communication’	266	‘performance’	49
		‘cyber’	49
		‘communication’	43
		‘blockchain’	42
		‘privacy’	42
		‘circuit’	42
‘fault_tolerance’	107	‘cyber_physical’	17
‘fault_injection’	78	‘machine_learning’	16
‘software_reliability’	57	‘anomaly_detection’	14
‘soft_error’	50	‘fault_tolerance’	13
‘web_service’	35	‘cloud_computing’	12
‘safety_critical’	34	‘fault_injection’	11
‘error_detection’	33	‘control_flow’	8
‘fault_detection’	32	‘test_case’	5
‘control_flow’	31	‘soft_error’	5
‘ad_hoc’	31	‘virtual_machine’	4
		‘smart_grid’	4
		‘processing_element’	4
		‘safety_critical’	4
		‘clock_synchronization’	3
		‘test_suite’	3
